# Values clarification workshops to improve abortion knowledge, attitudes and intentions: a pre-post assessment in 12 countries

**DOI:** 10.1186/s12978-018-0480-0

**Published:** 2018-03-05

**Authors:** Katherine L. Turner, Erin Pearson, Allison George, Kathryn L. Andersen

**Affiliations:** 1 Global Citizen, LLC Consulting, 732 Ninth St., No. 521, Durham, 27705 NC USA; 2 UNC-Chapel Hill Gillings School of Global Public Health, Chapel Hill, USA; 3 Public Health Solutions, 40 Worth Street, 5th Floor, New York, 10013 NY USA; 4 Technical Innovation and Evidence, Ipas, P.O. Box 9990, Chapel Hill, 27515 NC USA

**Keywords:** Values clarification, Attitude transformation, Abortion, Stigma

## Abstract

**Background:**

Women’s access to abortion care is often denied or hampered due to a range of barriers, many of which are rooted in abortion stigma. Abortion values clarification and attitude transformation (VCAT) workshops are conducted with abortion providers, trainers, and policymakers and other stakeholders to mitigate the effects of abortion stigma and increase provision of and access to abortion care. This study assesses changes in knowledge, attitudes, and behavioral intentions of VCAT workshop participants.

**Methods:**

Pre- and post-workshop surveys from 43 VCAT workshops conducted in 12 countries in Asia, Africa, and Latin America between 2006 and 2011 were analyzed to assess changes in three domains: knowledge, attitudes and behavioral intentions related to abortion care. A score was created for each domain (range: 0-100), and paired t-tests or Wilcoxon matched-pairs signed-ranks tests were used to test for significant differences between the pre- and post-workshop scores overall and by region and participant type (providers, trainers, and policymakers/other stakeholders). We also assessed changes in pre- and post-workshop scores for participants with the lowest knowledge and negative attitudes on the pre-workshop survey.

**Results:**

Overall, the mean knowledge score increased significantly from 49.0 to 67.1 (*p* < 0.001) out of a total possible score of 100. Attitudes and behavioral intentions showed more modest, but still statistically significant improvements between the pre- and post-workshop surveys. The mean attitudes score increased from 78.2 to 80.9 (p < 0.001), and the mean behavioral intentions score rose from 82.2 to 85.4 (*p* = 0.03). Among participants with negative attitudes pre-workshop, most shifted to positive attitudes on the post-workshop survey, ranging from 35.2% who switched to supporting unrestricted access to second-trimester abortion to 90.9% who switched to feeling comfortable working to increase access to contraceptive services in their country. Participants who began the workshop with the lowest level of knowledge experienced the greatest increase in mean knowledge score from 20.0 to 55.0 between pre- and post-workshop surveys (*p* < 0.001).

**Conclusions:**

VCAT workshop participants demonstrated improvements in knowledge, attitudes, and behavioral intentions related to abortion care. Participants who entered the workshops with the lowest levels of knowledge and negative attitudes had the greatest gains in these domains.

## Plain English summary

Women are often unable to access safe abortion services due to abortion stigma, which prevents potential abortion providers from offering abortion services and prevents other decision-makers such as policymakers or community leaders from supporting abortion service provision. This study found that abortion values clarification and attitude transformation (VCAT) workshops improve participants’ knowledge and attitudes about abortion as well as their intentions to support abortion care, especially among those who come to the workshops with the least knowledge and most negative attitudes.

## Background

Globally, an estimated 22 million unsafe abortions occur each year, resulting in the preventable deaths of 47,000 women annually [1]. Nearly all unsafe abortions occur among women in developing countries [2]. The determinants of safety for an induced abortion, such as method used and gestational age, are greatly influenced by underlying social factors, including the legal context, the availability services, levels of stigma, women’s access to information and women’s age and socioeconomic status [3]. Young, poor, rural and indigenous women often have the least access to high-quality abortion care and suffer the most negative consequences; 41% of unsafe abortions in developing regions are among young women aged 15–24 years [4].

Stigma is a learned behavior that impacts provision of and access to abortion care and the social environment surrounding it. Research suggests that abortion care providers in sub-Saharan Africa and Southeast Asia face personal conflicts, stigmatization and victimization with regards to delivering abortion care because of negative attitudes belonging to family, community and policymakers as well as their colleagues [5]. The World Health Organization recommends use of values clarification interventions as an integral part of training for abortion providers [6] to address abortion stigma as a root cause of barriers to abortion service delivery. A range of other stakeholders, influenced by their values, beliefs, attitudes and biases, may also impede women’s right to access abortion care. Policymakers and law enforcement impact abortion availability and accessibility through the language, passage, interpretation and enforcement of laws and policies. Ministry of health officials develop service delivery standards and guidelines that outline how and by whom services will be delivered at each level of the health system and the roles, responsibilities and limitations of health system administrators, providers and other health workers. These standards and guidelines may include restrictions that are not required by law or medically necessary that impede women’s access to care. All of these stakeholders possess and act upon values and attitudes which may be guided by misinformation and unexamined, internalized social norms and mores against abortion rather than factually-correct information and a belief in women’s right to abortion or an understanding of how restricting access to abortion increases women’s risk of death and disability.

### Values clarification

Values clarification (VC) is the process of examining one’s basic moral reasoning [7] to identify the values that one finds most meaningful and important [8]. The process can help an individual (1) identify when these core values conflict with assumptions or actions that may be informed by social norms and other external influences and (2) examine alternate values and their consequences. Although there is little published literature evaluating strategies for changing attitudes and behaviors of health care providers or other stakeholders [9–13], evidence supports the use of VC principles to improve attitudes and behaviors for social and health issues [14, 15]. In the field of sexual and reproductive health, VC has been employed to reduce HIV stigma [16], aid the integration of medical abortion into health care facilities [17, 18] and increase support for abortion care [19–23].

Use of VC to increase support for abortion services has yielded success in South Africa as shown in a study by Dickson-Tetteh & Reese. An evaluation of over 4000 providers who participated in such workshops demonstrated that close to 70% said that the workshop was helpful in strengthening their ability to work with abortion clients compared to before the workshop [24]. Interviews with a variety of stakeholders who participated in 3-day VC workshops in Limpopo, South Africa indicated that 93.2% of participants expressed increased compassion for women seeking abortions and the clinicians providing services [19]. Another study examining a three-part VC exercise used with 34 nursing students in South Africa found that the number of participants who were against abortion at the start of the training decreased by almost half; additionally, three of the five participants who were originally in favor of abortion only under specific circumstances decided they were not in a position to judge those seeking services upon completion of the exercise [23].

### Abortion values clarification and attitude transformation (VCAT)

Abortion values clarification and attitude transformation (VCAT) is an intervention that is grounded in values theory [7] and the Transtheoretical Model [25–27] and builds on similar interventions in other fields [28]. Abortion VC was first implemented in South Africa [19] and then developed by Turner into a global VCAT toolkit and strategy [28]. In VCAT interventions, trained facilitators lead diverse stakeholders through a process conducted in an emotionally safe environment in which they examine their personal values, attitudes and actions related to abortion; engage in honest, open-minded and critical reflection and evaluation of personally-relevant abortion information and situations and fully comprehend the harmful consequences of stigmatizing abortion and restricting service delivery and access to care. In abortion VCAT workshops, participants:

- Challenge deeply-held assumptions and myths
- Clarify and affirm their values and potentially resolve values conflicts
- Potentially transform their beliefs and attitudes that impact behaviors
- State their intentions to act in accordance with their affirmed values

Through this process, VCAT addresses some of the root causes of stigma-related barriers to abortion service delivery, access and quality.

**Fig. 1:**
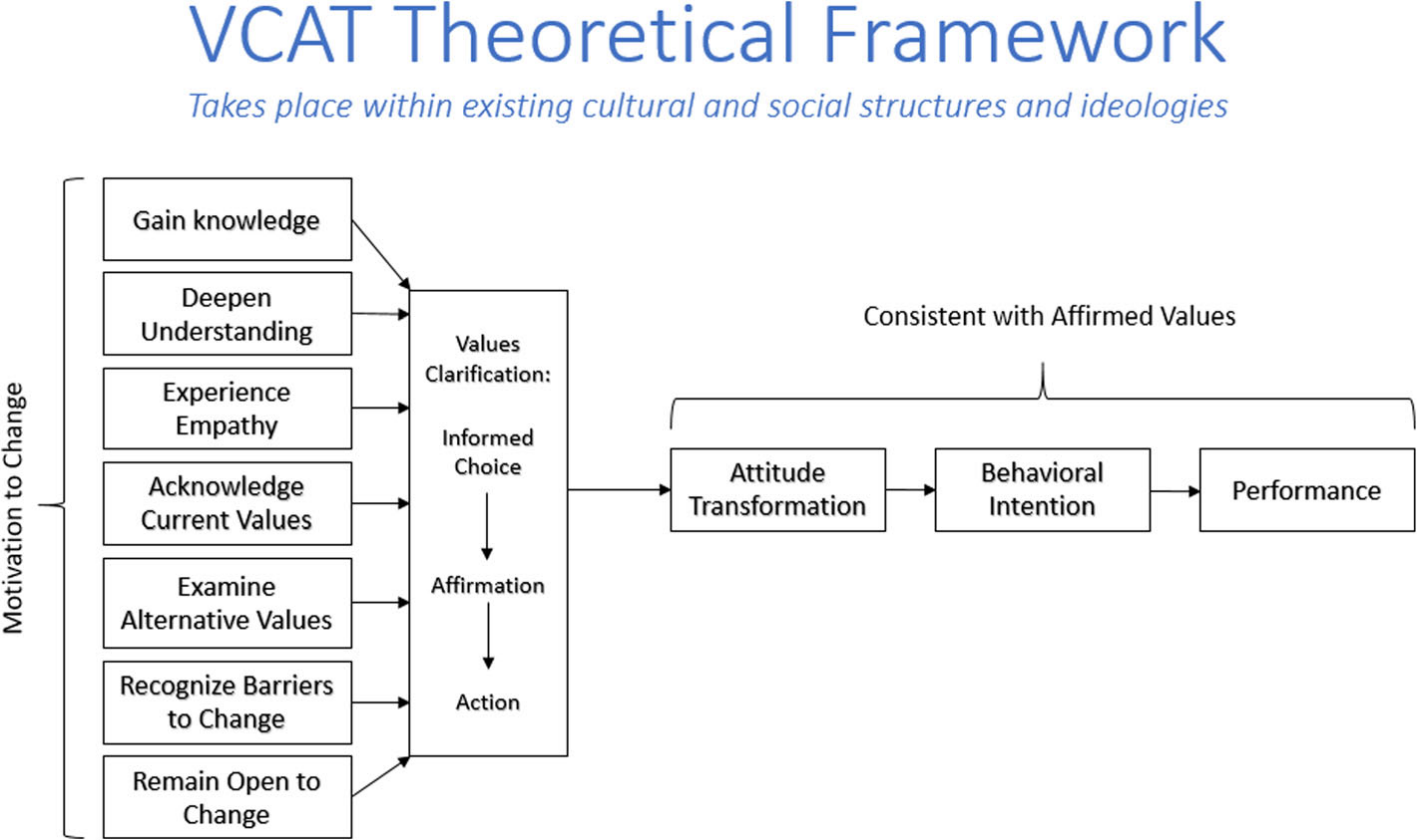
Abortion VCAT theoretical framework. Figure was published on p.6 of the VCAT Toolkit [28] and reproduced here with permission from Ipas

The VCAT theoretical framework developed by Turner and Chapman Page [28] posits that values play a critical role in determining how people make decisions and ultimately act (Fig. 1). The abortion VCAT process takes place within existing cultural and social structures and norms, which are extremely influential in shaping people’s attitudes and values. As Dewey states [29], “Valuing occurs when the head and the heart…unite under the direction of action.” Values tend to have persistence, assume a pattern in our lives and impact our *attitudes* and behaviors. This framework places the process of VC within a larger context of attitude transformation, behavioral intentions and, ultimately, behavior or performance. This is unlike traditional VC, in which the end goal is clarified values.

The framework begins with the willingness to change; people must be open to examining their values and potentially changing their attitudes and practices. Participants who effectively engage in the abortion VCAT process: gain new knowledge, deepen their understanding of existing or new knowledge, experience empathy for people who seek, provide or are affected by abortion, clarify current values on abortion, explore alternative values, recognize barriers to change, and remain open to change. Turner and Chapman Page [28] modified the three main stages of values clarification: making an informed value choice, affirming that choice, and acting on the chosen value, which reflects the process and cognitions an individual would go through when thoughtfully choosing among competing alternatives and deciding on a particular course of action. The framework hypothesizes that, after undergoing the values clarification process, participants’ attitudes are expected to be consistent with their clarified, affirmed values. Attitudes and beliefs influence behavioral intentions, which in turn predict behaviors [25–27]. These constructs of personal attitude and behavioral intention have been successful in predicting health workers’ behaviors in several studies [30, 31].

### The VCAT strategy and activities

Strategies based on the abortion VCAT framework focus on the real consequences of abortion stigma: unsafe abortion, which can result in women’s injury or death. Faced with these potentially dire consequences, participants in the VCAT process often move along a progressive continuum from obstruction to tolerance to acceptance to provision or support, and for some, to advocacy for high-quality, comprehensive abortion care for all women. The VCAT intervention consists of participatory presentations and activities (14 total activities in the Toolkit) that engage participants with accurate abortion information, realistic scenarios, critical self-reflection, empathy-evoking experiences and meaningful dialogue on abortion beliefs, values and professional ethics and responsibilities. Each activity includes specific, timely, measureable and detailed learning objectives for participants. Activities are intended to cultivate attitudes that are supportive of women’s right to safe abortion care. Participants are given vignettes to discuss and asked to consider the health and social implications when policymakers, providers and other stakeholders restrict access to safe abortion services for certain women. VCAT workshops employ adult learning principles and methodologies, including large and small group discussion, expressive activities, case studies, individual and group work, personal journals and interviews, and self-analysis worksheets. VCAT workshops were designed for use with diverse audiences. Additional versions of the VCAT toolkit activities have been developed to specifically address second trimester abortion, medical abortion, young women’s needs and perspectives on abortion care, and sex selective abortion as well as other health areas, including contraceptive services and respectful maternity care.

The present study sought to assess whether VCAT workshops implemented by Ipas, an international NGO seeking to reduce unsafe abortion and increase women’s access to comprehensive abortion care, improved participants’ knowledge, attitudes and behavioral intentions pertaining to abortion care.

## Methods

### Study design

This study used matched pre- and post-workshop surveys to assess changes in three domains: knowledge, attitudes and behavioral intentions related to abortion care. During the study period of 2006 to 2011, 118 VCAT workshops were reported, but only 46 of those workshops (39%) conducted matched pre- and post-surveys with participants. The other workshops either did not conduct the pre- and post-workshop surveys, or no code was used to link the pre-workshop survey to a participant’s post-workshop survey. In addition, data from three workshops were excluded due to excessive missing data. This analysis presents findings for the convenience sample of 43 workshops in Africa (*n* = 22), Asia (*n* = 18), and Latin America (*n* = 3), which included a total of 641 participants with matched pre- and post-workshop surveys. The participants in workshops included in this study comprise three main groups: 1) stakeholders, including elected and ministry of health officials, lawyers, journalists, and other political and community leaders; 2) trainers attending training-of-trainer (TOT) events to become trainers on abortion clinical skills; and 3) health care providers, including physicians and mid-level providers such as midwives and nurses. Workshop participants were typically self-nominated or selected based on a perceived or assessed willingness to support, provide or advocate for abortion care, reflecting the reality of conducting interventions such as VCAT.

### Data

Standardized pre- and post- workshop surveys were administered to all participants in each of the workshops included in this analysis. Questions were developed in conjunction with the VCAT curriculum to ensure that the measures matched the content of the workshops. The questionnaires were developed in English, then translated and back-translated into both Spanish and French to ensure consistency across country settings. Overall, the survey aimed to measure changes in participants’ knowledge, attitudes and behavioral intentions related to abortion care.

Facilitators were able to modify the standard questionnaires to better reflect their country setting. As a result, not all workshops’ surveys included all of the standardized components. In particular, all (43 workshops, 641 participants) included the attitude component, 39 workshops (564 participants) included the knowledge component, and 32 workshops (471 participants) included the behavioral intentions component. The pre-workshop survey was completed at the beginning of each workshop, and the post-workshop survey was completed at the end. Data were entered using EpiData software.

### Measures

Three domains were assessed by the survey: knowledge, attitudes, and behavioral intentions. The number of items assessing each domain varied somewhat across workshops, and to account for this, scores on a scale of 1 to 100 were created for each of the three domains. Knowledge questions evaluated the participants’ knowledge about abortion laws in their country, safe abortion methods, consequences of unsafe abortion, and statistics on unsafe abortion in their country. Knowledge scores were calculated by dividing the number of correct responses by the total number of items (20 on the standard survey), and multiplying by 100. Attitude questions were asked on a five-point Likert scale and evaluated the participants’ comfort with the topic of abortion, including their willingness to discuss abortion with colleagues, family and friends, attitudes toward the legality and provision of abortion in their setting. Attitude scores were calculated by summing the five-point responses, dividing by five times the total number of items (18 on the standard survey), and multiplying by 100. Behavioral intention questions evaluated the participants’ intent to participate in activities such as information sharing and advocacy for abortion and, among clinical providers, intentions to perform or assist with abortion procedures in the next six months. Behavioral intention scores were calculated by dividing the number of positive responses by the total number of items (7 on the standard survey), and multiplying by 100.

### Missing data

Missing data were handled in two ways. For the knowledge and behavioral intentions components, items that were left unanswered were conservatively coded as “incorrect” or “no, does not intend”, respectively. On the knowledge section it was assumed that a participant did not know the correct answer to the question if she or he left the question unanswered. Similarly, on the behavioral intentions section it was assumed that the participant did not intend to participate in the activity or was unsure of whether they would participate in the activity if she or he did not answer the question.

Missing data in the attitudes section could not be easily recoded because attitudes were measured using scales. Missing data were considered to be “not missing at random” since leaving an item blank, such as agreement with second trimester abortion, was expected to be related to the unobserved value. Multivariate multiple imputation was used to assign values for each missing item under the attitude domain using the iterative Markov chain Monte Carlo (MCMC) method [32].

### Analysis

Data are presented for each domain overall and by workshop participant type (stakeholder, trainer or provider) and by region (Africa, Asia or Latin America). The mean score and associated standard deviation (SD) are presented for the knowledge and behavior scores, while the mean and standard error (SE) are presented from the multiple imputation sample for attitude scores. Paired t-tests were used to test for significant differences between the pre- and post-workshop scores where the sample size was sufficient to assume a normal distribution. For analyses with small sample sizes, such as for Latin America, the non-parametric Wilcoxon matched-pairs signed-ranks test was used to test for significant differences between the pre- and post-workshop scores.

We also assessed whether changes in the pre- and post-workshop knowledge scores varied based on participants’ knowledge levels at the beginning of the workshop. The pre-workshop scores were divided into quartiles, and within each quartile of the pre-workshop score, the mean pre- and post-workshop scores were calculated. Differences between the pre- and post-workshop scores within each quartile of the pre-workshop score were assessed using the paired t-test.

Finally, we present an analysis of changes in attitudes between the pre- and post-workshop surveys among participants with negative pre-workshop attitudes. This analysis presents data by question and was restricted to the 317 participants who received the 18 standard attitudes questions. For each question, participants’ attitudes were classified in three categories: 1) negative, “strongly disagree” or “disagree”; 2) neutral, “neither disagree nor agree”; or 3) positive, “agree” or “strongly agree.” Among those who were classified as negative on the pre-workshop survey, we present the proportion classified as negative, neutral and positive on the post-workshop survey. The Kruskal-Wallis one-way analysis of variance test, the non-parametric version of the one-way analysis of variance (ANOVA), was used to test for differences between the pre- and post-workshop surveys. Statistical significance was assessed at an alpha level of 0.05 for all analyses*.* Statistical analyses were conducted using Stata version 11.2.

## Results

Table 1 presents the mean scores for each domain overall and by workshop participant type. Overall, the mean knowledge score increased from 49.0 (SD = 23.5) on the pre-workshop survey to 67.1 (SD = 19.4) on the post-workshop survey (*p* < 0.001) (Table 1). Trainers had the highest mean pre-workshop knowledge score (mean = 68.1; SD = 17.9), which increased by almost 10 points on the post-workshop survey (*p* < 0.001). Among stakeholders, the mean score increased from 54.3 (SD = 19.4) on the pre-workshop survey to 68.1 (SD = 17.4) on the post-workshop survey (*p* < 0.001). Though providers had the lowest mean pre-workshop knowledge score (36.7), they also had the largest increase between the pre- and post-workshop surveys; the mean score increased by almost 25 points between the pre- and post-workshop surveys (*p* < 0.001).

**Table 1:** Mean pre- and post-workshop knowledge, attitude and behavioral intention scores, overall and by type of participants

	Knowledge						Attitudes						Behavioral Intentions					
		Pre-workshop		Post-workshop				Pre-workshop		Post-workshop				Pre-workshop		Post-workshop		
	n	Mean	(SD)	Mean	(SD)	*p*-value	n	Mean	(SE)^1^	Mean	(SE)^1^	*p*-value	n	Mean	(SD)	Mean	(SD)	*p*-value
Providers	300	36.7	(19.4)	60.9	(20.2)	< 0.001	332	77.9	(0.71)	81.6	(0.62)	< 0.001	162	81.6	(27.0)	88.1	(22.1)	0.007
Trainers	167	68.1	(17.9)	77.8	(13.7)	< 0.001	167	80.1	(0.93)	83.1	(0.76)	0.001	167	86.3	(26.4)	91.5	(17.7)	0.007
Stakeholders	97	54.3	(19.4)	68.1	(17.4)	< 0.001	142	77.4	(0.71)	76.7	(0.82)	0.177	142	77.9	(30.3)	75.0	(36.0)	0.374
Total	564	49.0	(23.5)	67.1	(19.4)	< 0.001	641	78.2	(0.47)	80.9	(0.43)	< 0.001	471	82.2	(28.0)	85.4	(26.8)	0.027

^1^Standard error (SE) is presented for the attitudes scores rather than the standard deviation (SD) because the mean for this score was derived from the multiple imputation sample

^2^*P*-values associated with paired t-tests

Overall, attitudes showed modest but statistically significant improvement between the pre- and post-workshop surveys from 78.2 (SE = 0.47) on the pre-workshop survey to 80.9 (SE = 0.43) on the post-workshop survey (p < 0.001). The largest increases were observed for providers whose mean attitude score increased by 4 points from 77.9 (SE = 0.71) on the pre-workshop survey to 81.6 (SE = 0.62) on the post-workshop survey (p < 0.001) (Table 1). A statistically significant increase in the attitudes score was also observed for trainers, from a mean of 80.1 (SE = 0.93) on the pre-workshop survey to 83.1 (SE = 0.76) on the post-workshop survey (*p* = 0.001). Statistically significant changes were not observed for stakeholders.

Similarly, behavioral intentions showed modest gains between the pre- and post-workshop surveys from 82.2 (SD = 28.0) on the pre-workshop survey to 85.4 (SD = 26.8) on the post-workshop survey (*p* = 0.027). Providers’ mean behavioral intentions score increased by almost 7 points from a mean of 81.6 (SD = 27.0) on the pre-workshop survey to 88.1 (SD = 22.1) on the post-workshop survey (*p* = 0.007) (Table 1). Among trainers, mean behavioral intention scores increased by 4 points from 86.3 (SD = 26.4) on the pre-workshop survey to 91.5 (SD = 17.7) on the post-workshop survey (p = 0.007). Again, no statistically significant changes were observed for stakeholders.

Table 2 presents the mean scores for each domain by region. Statistically significant improvements were observed in knowledge, attitudes and behavioral intentions for the participants in Africa. The mean knowledge score increased by almost 17 points between the pre- and post-workshop surveys (*p* < 0.001), and the mean attitudes score increased by 3 points (*p* < 0.001). The mean behavioral intentions score increased from 83.0 (SD = 28.6) on the pre-workshop survey to 86.8 (SD = 26.5) on the post-workshop survey (*p* = 0.030). In Asia there were significant increases in knowledge and attitude scores, but not in the behavioral intentions score. The mean knowledge score increased by 20 points, from 43.7 (SD = 24.3) on the pre-workshop survey to 63.7 (SD = 21.2) on the post-workshop survey (*p* < 0.001). The mean attitude score showed a more modest increase from 78.3 (SE = 0.72) on the pre-workshop survey to 81.0 (SE = 0.66) on the post-workshop survey (*p* = 0.001). In Latin America, statistically significant changes were not observed in any of the domains.

**Table 2:** Mean pre- and post-workshop knowledge, attitude and behavioral intention scores, overall and by region

	Knowledge						Attitudes						Behavioral Intentions					
		Pre-workshop		Post-workshop				Pre-workshop		Post-workshop				Pre-workshop		Post-workshop		
	n	Mean	(SD)	Mean	(SD)	*p*-value^2^	n	Mean	(SE) ^1^	Mean	(SE) ^1^	*p*-value^2^	n	Mean	(SD)	Mean	(SD)	*p*-value^2^
Africa	275	54.0	(21.7)	70.9	(17.0)	< 0.001	320	78.3	(0.67)	81.5	(0.59)	< 0.001	320	83.0	(28.6)	86.8	(26.5)	0.030
Asia	279	43.7	(24.3)	63.7	(21.2)	< 0.001	279	78.3	(0.72)	81.0	(0.66)	0.001	109	80.2	(28.4)	81.8	(28.3)	0.636
Latin America	10	60.5	(11.4)	61.0	(12.6)	0.907	42	77.0	(1.48)	76.7	(1.62)	0.882	42	80.9	(22.0)	84.1	(24.1)	0.147
Total	564	49.0	(23.5)	67.1	(19.4)	< 0.001	641	78.2	(0.47)	80.9	(0.43)	< 0.001	471	82.2	(28.0)	85.4	(26.8)	0.027

^1^Standard error (SE) is presented for the attitudes scores rather than the standard deviation (SD) because the mean for this score was derived from the multiple imputation sample

^2^*P*-values associated with paired t-tests or Wilcoxon matched-pairs signed-ranks test, as appropriate

**Fig. 2:**
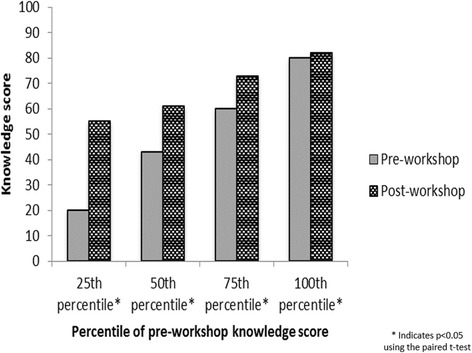
Change in mean knowledge score between pre- and post-workshop surveys by quartile of the pre-workshop knowledge score (*n* = 564). Figure was created by the authors

Figure 2 presents a comparison of the mean pre- and post-workshop knowledge scores by quartile of the pre-workshop knowledge score. Increases in the mean knowledge score were observed between the pre- and post-workshop surveys for all participants, regardless of pre-workshop knowledge. However, the participants who began the workshop with the lowest level of knowledge, those in the 25th percentile, experienced the greatest increase in mean knowledge score (35 points) between the pre- and post-workshop surveys (*p* < 0.001). Participants in the 50th and 75th percentiles of the pre-workshop knowledge score saw similar increases in mean score of 18 points (*p* < 0.001) and 13 points (*p* < 0.001), respectively. A smaller increase in mean score was observed for participants in the highest quartile of the pre-workshop knowledge score (2 points; *p* = 0.04). Similar, statistically-significant results were found for attitudes and behavioral intentions; those who reported more negative attitudes and behavioral intentions on the pre-workshop survey showed the largest gains in mean score on the post-workshop survey (data not shown).

Table 3 presents an analysis of the post-workshop attitudes among those who had negative attitudes on the pre-workshop survey. Among the 317 participants who completed the standardized attitudes questions, the number who had negative attitudes pre-workshop ranged from 9 (2.8%) who did not support provision of family planning in their country to 156 (49.2%) who felt that access to second trimester abortion services should be restricted to certain circumstances. Most participants who had negative attitudes pre-workshop reported a shift to positive attitudes on the post-workshop survey. Of particular interest were attitudes about support for abortion services as permitted by law; of the 47 participants who were unsupportive pre-workshop, 31 (66.0%) reported positive attitudes, 8 (17.0%) reported neutral attitudes, and 8 (17.0%) maintained their negative attitudes on the post-workshop survey (*p* = 0.010). Similarly, 41 participants reported that they were uncomfortable with working to increase access to abortion services pre-workshop, and the majority (63.4%) reported positive attitudes on the post-workshop survey (*p* < 0.001). Comfort with performing or assisting with an abortion procedure showed a smaller improvement; of the 41 who reported being uncomfortable pre-workshop, 17 (41.5%) reported positive attitudes, 8 (19.5%) reported neutral attitudes, and 16 (39.0%) maintained their negative attitudes on the post-workshop survey (*p* < 0.001). Support for access to safe, comprehensive abortion care increased for both the first and second trimester, but 36.4% of participants maintained their negative attitudes about second trimester care, compared to only 10.5% for first trimester care.

**Table 3:** Post-workshop attitudes among 317 participants with negative attitudes at pre-workshop survey

	Negative attitude at pre-test	Post-workshop attitudes among those who had negative attitudes at pre-test						
		Negative		Neutral		Positive		
Attitude question	n	n	(%)	n	(%)	n	(%)	*p*-value^1^
The issue of abortion is important to me	41	13	(31.7)	2	(4.9)	26	(63.4)	0.002
I support the provision of FP and contraceptive services in my country	9	1	(11.1)	0	(0)	8	(88.9)	0.909
I feel comfortable working to increase access to FP and contraceptive services in my country	11	1	(9.1)	0	(0)	10	(90.9)	0.419
I support the provision of abortion services as permitted by law in my country	47	8	(17.0)	8	(17.0)	31	(66.0)	0.010
I feel comfortable working to increase access to abortion services as permitted by law in my country	41	4	(9.8)	11	(26.8)	26	(63.4)	< 0.001
I feel comfortable talking with my closest friends about my involvement with abortion care	30	6	(20.0)	4	(13.3)	20	(66.7)	0.007
I feel comfortable talking with my closest family members about my involvement with abortion care	47	13	(27.7)	9	(19.1)	25	(53.2)	< 0.001
I would feel comfortable observing an abortion procedure^2^	41	13	(31.7)	9	(22.0)	19	(46.3)	< 0.001
I would feel comfortable performing or assisting an abortion procedure^2^	41	16	(39.0)	8	(19.5)	17	(41.5)	< 0.001
I am clear about my personal values concerning abortion	18	1	(5.6)	1	(5.6)	16	(88.8)	0.534
I do not feel conflicted about abortion	67	17	(25.4)	13	(19.4)	37	(55.2)	0.013
I can clearly explain my personal values concerning abortion	19	3	(15.8)	3	(15.8)	13	(68.4)	0.106
I can respectfully explain values concerning abortion that conflict with mine	22	3	(13.6)	2	(9.1)	17	(77.3)	0.276
I feel empathy for women who have experienced abortion	25	9	(36.0)	6	(24.0)	10	(40.0)	< 0.001
All women should have access to safe, comprehensive abortion care in the first trimester	38	4	(10.5)	12	(31.6)	22	(57.9)	0.002
Access to first-trimester abortion should not be restricted to certain circumstances	111	47	(42.3)	14	(12.6)	50	(45.1)	< 0.001
All women should have access to safe, comprehensive abortion care in the second trimester	88	32	(36.4)	16	(18.2)	40	(45.4)	< 0.001
Access to second-trimester abortion should not be restricted to certain circumstances	156	77	(49.4)	24	(15.4)	55	(35.2)	< 0.001

^1^P-value associated with the Kruskal-Wallis one-way analysis of variance test

^2^Excludes attendees of stakeholder workshops since most were non-clinical (*n* = 198)

## Discussion

This study documents the results of abortion VCAT workshops to improve knowledge, attitudes and intentions to provide support, assist or advocate for abortion care. Across workshop types and locations, the greatest improvements were observed in knowledge. Mean knowledge scores were low pre-workshop, especially among providers and participants from Asia, but these scores increased by up to 24 points between the pre- and post-workshop surveys. Participants who entered the workshops with the lowest levels of knowledge experienced the greatest gains. However, participants with the lowest pre-workshop knowledge did not catch up to their more knowledgeable peers; their post-workshop scores were still lower than those of participants who had high pre-workshop scores. Previous work has demonstrated a dose-response relationship between exposure to abortion messages and abortion knowledge, suggesting that multiple exposures to messages about abortion may be needed over time to increase knowledge in a meaningful way [33]. A question that could be further explored is whether the post-survey mean knowledge scores were sufficiently high, and given the relationship between knowledge and attitudes in the VCAT theoretical framework, whether higher knowledge gains might result in more positive attitudes.

More modest increases in the attitudes scores were observed, which was expected because attitudes may be less pliable than knowledge and is in line with other studies [34]. The VCAT workshops’ effect on participants’ attitudes varied by question. VCAT workshops were more successful in shifting those with negative attitudes pre-workshop to neutral or positive attitudes for questions that were less controversial, such as support for family planning. Meanwhile, attitudes about more stigmatized issues, such as second trimester abortion care, were not as readily improved, which is in line with other studies that have shown challenges in improving provider attitudes about reasons for abortion that may be considered more controversial in some settings [34]. At the community level, studies have shown that exposure to abortion messages is associated with positive attitudes about abortion [35], but more research is needed to understand what is required to achieve the tipping point from negative to positive attitudes about abortion. Though the increase in the mean attitude score was small, it is important that the majority of participants who had negative attitudes pre-workshop showed improved attitudes on the post-workshop survey. A goal of the VCAT workshops is to shift individuals along the continuum from obstructionist to tolerant to supportive attitudes. Those whose attitudes shift from negative to neutral may be less likely to obstruct and may even support or facilitate women’s access to care and colleagues’ service provision. In addition, individuals who leave VCAT workshops with positive attitudes may be the most likely to provide, support and advocate for abortion.

The smallest increases were observed in the behavioral intentions scores, which are partially explained by the very high scores on the pre-workshop survey. It is likely that the pre-workshop behavioral intentions scores were high because participants were either selected by NGO staff and colleagues due to past support for or work in abortion or because they meet other screening criteria for abortion VCAT workshops. Thus, many of the participants likely intended to support or provide abortion services prior to the VCAT workshop. This affects how much change is likely or possible between pre- and post-test. The data suggest that those who enter the workshops with the lowest levels of knowledge and more negative attitudes and behavioral intentions are the participants who experience the greatest gains in these domains. However, it is possible that even modest improvements in attitudes and behavioral intentions could affect workshop participants’ willingness to support or provide abortion services or improve the quality of care they provide, based on an improved understanding of women’s right to abortion and consequences of poor access to or quality of care. VCAT workshops and post-test scores can help organizations decide which participants are committed to abortion service provision and thus merit the significant investment of clinical training and follow-up support at their facilities.

Statistically significant increases were observed in all three domains for provider and trainer workshops, but stakeholder workshops only resulted in a statistically significant increase in knowledge. Abortion VCAT workshops were originally designed for use with abortion providers, but the stakeholder workshops were conducted with a diverse group of people, including politicians, journalists and community leaders. Results suggest that new VCAT toolkit modules may be needed to improve attitudes and behavioral intentions for non-clinician participants such as those who participate in stakeholder workshops.

Regional variation was observed in the results with statistically significant improvement across all three domains in Africa, in knowledge and attitudes in Asia, and in none of the domains in Latin America. In Asia, we did not observe statistically significant changes in behavioral intentions, which may be due to the smaller sample size for this domain compared to the knowledge and attitudes domains. Approximately 60% of the participants in Asia did not receive the behavioral intentions component of the pre- and post-workshop surveys, which resulted in a much smaller sample size for analysis of this domain (109 participants) compared to the knowledge and attitudes domains (279 participants). In Latin America, significant changes were not observed in any of the domains. The VCAT organizers and facilitators in this region attribute this to the survey instrument and maintain that a context-specific pre- and post-test is needed. More contextual analysis is needed to better understand the results of abortion VCAT workshops in Latin America.

### Limitations

This study had several limitations. The primary limitation of this study was that surveys were only conducted before and immediately following each workshop, and as a result, it was not possible to measure lasting change. Because identifiers were not recorded on the participants’ pre- and post-workshop surveys, the changes in the three domains assessed by the surveys could not be linked to behavioral outcomes such as abortion procedures performed or advocacy efforts. It is possible that better attitudes and behavioral intentions on the post-workshop survey reflect social desirability bias rather than a true improvement in attitudes and intentions, and though the results of this study suggest positive change, behavior change cannot be assessed. Future research could link changes in knowledge, attitudes and behavioral intentions with actual behaviors and practices.

Another limitation is selection bias. As previously mentioned, it is likely that pre-workshop behavioral intention scores were high because participants self-selected or were selected by workshop organizers to participate in the VCAT workshops because they had demonstrated support for abortion service provision and access in their previous work. Though selection bias should be recognized, it also represents a reality of implementing an intervention such as VCAT in that workshops would usually be conducted with willing participants. In addition, this study only reflects findings from a small convenience sample of all VCAT workshops. It is possible that the workshops for which data were available had more conscientious organizers and facilitators, which could be associated with more positive workshop outcomes. As a result, results may not be representative of all VCAT workshop participants.

Finally, missing data was a limitation. A conservative approach was taken, and missing data for the knowledge and behavioral intentions was coded as “incorrect” or “no”. As a result, the calculated scores may be an underestimate of the true knowledge and behavioral intentions scores.

## Conclusions

This study demonstrated that abortion VCAT workshops led to improvements in participant knowledge, attitudes, and behavioral intentions regarding abortion. Findings suggest that participants who enter the workshops with the lowest levels of knowledge and more negative attitudes are the participants who experience the greatest improvements in those domains. Additional research is needed to understand long-term gains in knowledge and attitudes resulting from VCAT as well as actual behavior change in support for or provision of abortion services.

## Abbreviations

ANOVA: Analysis of variance
NGO: Non-governmental organization
SD: Standard deviation
SE: Standard error
TOT: Training of the trainer
VC: Values clarification
VCAT: Values clarification and attitude transformation
